# Expected value of information in the case of mixed strategies

**DOI:** 10.1186/1745-6215-14-S1-P51

**Published:** 2013-11-29

**Authors:** Gianluca Baio

**Affiliations:** 1 University College London, London, UK

## 

Cost-effectiveness analysis (CEA) represents the most important tool in the health economics literature to quantify and qualify the reasoning behind the optimal decision process in terms of the allocation of resources to a given health intervention. However, the practical application of CEA in regulatory process is often limited by some critical barriers, and decisions in clinical practice are frequently influenced by factors that do not contribute to an efficient resources allocation, leading to inappropriate drug prescription and utilisation. Moreover, most of the times there is uncertainty about the real cost-effectiveness profile of an innovative intervention, with the consequence that it is usually impossible to obtain an immediate and perfect substitution of an intervention with another having a better cost-effectiveness ratio. We propose a rational approach to CEA within regulatory processes basing our analysis in a Bayesian decision-theoretic framework and proposing an extension of the application of well known tools (such as the expected value of information) to such cases. The regulator can use these tools to identify the economic value of reducing the uncertainty surrounding the cost-effectiveness profile of the several alternatives. This value can be compared to the one that is generated by the actual combination (in terms of market shares) of different options: one that is the most cost-effective and others in the same therapeutic category that, despite producing clinical benefits, are less cost-effective. We use prescription of statins as a case study.

